# Recombinant *Clostridium difficile* Toxin Fragments as Carrier Protein for PSII Surface Polysaccharide Preserve Their Neutralizing Activity

**DOI:** 10.3390/toxins6041385

**Published:** 2014-04-22

**Authors:** Maria R. Romano, Rosanna Leuzzi, Emilia Cappelletti, Marta Tontini, Alberto Nilo, Daniela Proietti, Francesco Berti, Paolo Costantino, Roberto Adamo, Maria Scarselli

**Affiliations:** 1 Novartis Vaccines, Via Fiorentina 1, Siena 53100, Italy; E-Mails: maria_rosaria.romano@novartis.com (M.R.R.); rosanna.leuzzi@novartis.com (R.L.); marta.tontini@novartis.com (M.T.); alberto.nilo@novartis.com (A.N.); daniela.proietti@novartis.com (D.P.); francesco.berti@novartis.com (F.B.); paolo.costantino@novartis.com (P.C.); roberto.adamo@novartis.com (R.A.); 2 Novartis Vaccines Institute for Global Health, Via Fiorentina 1, Siena 53100, Italy; E-Mail: emilia.cappelletti@novartis.com

**Keywords:** *Clostridium difficile*, glycoconjugate, PSII polysaccharide, recombinant toxin fragment

## Abstract

*Clostridium difficile* is a Gram-positive bacterium and is the most commonly diagnosed cause of hospital-associated and antimicrobial-associated diarrhea. Despite the emergence of epidemic *C. difficile* strains having led to an increase in the incidence of the disease, a vaccine against this pathogen is not currently available. *C. difficile* strains produce two main toxins (TcdA and TcdB) and express three highly complex cell-surface polysaccharides (PSI, PSII and PSIII). PSII is the more abundantly expressed by most *C. difficile* ribotypes offering the opportunity of the development of a carbohydrate-based vaccine. In this paper, we evaluate the efficacy, in naive mice model, of PSII glycoconjugates where recombinant toxins A and B fragments (TcdA_B2 and TcdB_GT respectively) have been used as carriers. Both glycoconjugates elicited IgG titers anti-PSII although only the TcdB_GT conjugate induced a response comparable to that obtained with CRM_197_. Moreover, TcdA_B2 and TcdB_GT conjugated to PSII retained the ability to elicit IgG with neutralizing activity against the respective toxins. These results are a crucial proof of concept for the development of glycoconjugate vaccines against *C. difficile* infection (CDI) that combine different *C. difficile* antigens to potentially prevent bacterial colonization of the gut and neutralize toxin activity.

## 1. Introduction

*Clostridium difficile* is a Gram-positive, spore-forming and toxin-producing anaerobic gastrointestinal pathogen that is the major cause of antibiotic-associated colitis. *C. difficile* has been isolated from several domestic and nondomestic animal species, and has been associated with diarrhea in horses, pigs, dogs and cats. In humans, *C. difficile* associated diarrhea (CDAD) is the most commonly diagnosed cause of hospital-associated and antimicrobial-associated diarrhea [1].

*C. difficile* infection (CDI) has grown tremendously since 1978, and over the last decade, the incidence and severity of CDI has increased significantly and affected new patient groups. Today, the disease represents a major social and economic burden [2]. Since 2005, CDI has been increasingly reported among young, healthy individuals residing in the community. An estimated 20% to 28% of CDI is community associated with an incidence of 20 to 50 cases per 100,000 population in the United States, Sweden and England [3].

At the moment, there is no vaccine against *C. difficile*, despite the increase in the incidence of the disease observed in the last decades [4,5].

The virulence of *C. difficile* is conferred primarily by two large exotoxins, toxins A and B, and there is evidence that protection against severe CDI is mediated by systemic antibodies to TcdA and TcdB [6,7,8]. Both toxins present three distinct functional domains: an N-terminal enzymatic domain consisting of glucosyl-transferase (GT) and cysteine protease (CP) moieties, a central translocation (T) domain that mediates import into host cells and a C-terminal receptor binding domain (RBD) with 38 tandem repeats [9].

Although a number of studies have demonstrated that anti-toxin circulating antibodies are effective in the treatment of severe CDI [10,11], supporting the key role of toxin immunity in preventing the lethal outcome of this infection, the use of toxoid-based vaccines in humans has been limited for a long time. Recently, preparations of formaldehyde-inactivated toxoid from *C. difficile* culture supernatants have been able to confer protective immunity in clinical trials [11,12,13,14].

To overcome the safety issues potentially associated to the large-scale production of toxoids, such as exposure to toxins and spores, the use of recombinant proteins has been proposed as an attractive alternative for development of vaccines against CDAD [15]. Several studies have demonstrated the ability of recombinant toxin fragments to induce robust immunity against lethal challenge with *C. difficile*. In particular, TcdA and TcdB RBDs, cloned and purified from a variety of hosts, have been proven to induce both systemic and mucosal neutralizing antibodies in animal models [16,17,18]. Our group has recently shown that co-administration of a cell binding domain fragment of TcdA (TcdA_B1) and the glucosyltransferase moiety of TcdB (TcdB_GT) can induce systemic IgGs, neutralizing both the respective toxins and protecting vaccinated animals from death in hamster animal model of lethal infection. The presence of anti-TcdA and TcdB antibodies was assessed in gut contents, suggesting that systemic vaccination with this pair of recombinant polypeptides can limit the disease caused by toxin production during CDI [19]. However, anti-toxins antibodies elicited by toxin fragments are not able to limit the level of bacterial load in the gut [20].

Recently, it has been shown that *C. difficile* vegetative cells express three highly complex polysaccharides on their cell surface, named PSI, PSII and PSIII. Among those three carbohydrates, PSII has been found to be the more abundantly expressed by the hypervirulent rybotype O27 [21]. The PSII is a polysaccharide composed of a hexaglycosyl phosphate repeating unit [-6)-β-d-Glcp-(1-3)-β-d-GalpNAc-(1-4)-α-d-Glcp-(1-4)-[β-d-Glcp-(1-3)]-β-d-GalpNAc-(1-3)-α-d-Manp-(1-P] [22].

We have previously employed the high-resolution magic angle spinning (HR-MAS) NMR on vegetative whole cells from a collection of clinical isolates and have detected PSII on the surface of different rybotypes, such as 001, 018, 027, 078 and 126 [23]. The list of isolates analyzed by this technique has been further updated, detecting PSII in a number of clinical and environmental isolates, including strain 630 [24]. Therefore, PSII is as a surface antigen conserved among the most common strains and can represent a relevant target for the development of a carbohydrate-based vaccine.

In confocal microscopy, examination of vegetative cells using anti-PSII antibodies revealed that PSII does not appear as a typical thick and even bacterial capsule; then it can be hypothesized that the polysaccharide is present either as cell wall-linked polysaccharide not bound to peptidoglycan or as a conjugate with lipoteichoic acids [21,24].

Interestingly, strain 630 and the hypervirulent strain R20291 can form *in vitro* structured biofilms, where the presence of PSII could be detected by antibodies against the phosphorylated hexaglycosyl repeating unit [25]. This suggests that extracellular PSII could play a role in determining the biofilm’s architecture of *C. difficile* as component of extracellular matrix.

Glycans are T cell independent antigens, but they can be turned into molecules able to evoke a T cell memory response following conjugation to a carrier protein [26]. Anti unconjugated PSII IgM antibodies have been generated in pregnant pigs vaccinated with a non-adjuvanted PSII containing an average of six repeating units [27]. PSII, after conjugation to CRM_197_ (non-toxic mutant of diphtheria toxin) [28], a carrier protein widely used for the manufacturing of glycoconjugate vaccines [29], was formulated with the adjuvant MF59 and tested in Balb/C mice, inducing high levels of specific anti carbohydrate IgG, a class of antibodies which is generally relevant to induce protection against the sugar coated pathogens [23]. Therefore, conjugation of PSII to the carrier protein could ensure, as expected, the IgM-to-IgG switch. Noteworthy, glycoarray analysis has demonstrated that specific IgA antibodies in the stool of patients infected with *C. difficile* can recognize the nonphosphorylated PSII hexasaccharide hapten, suggesting that under exposure to PSII the human immune system may furnish a mucosal response against carbohydrate epitopes from PSII [30].

The co-administration of multiple *C. difficile* antigens, by using recombinant toxin fragments conjugated to PSII could have the potential to prevent colonization and protect against *C. difficile* disease. We envisaged in conjugation of PSII to toxins as a strategy to ensure co-delivery of the two antigens, using the toxin as carrier protein for the polysaccharide. With this aim, we have evaluated the immunological response of PSII-toxin based glycoconjugates in mouse, investigating the possible double role of the two TcdA_B2 and TcdB_GT fragments, as carrier protein for the PSII polysaccharide and antigens able to elicit antibodies with toxin neutralizing activity.

## 2. Results and Discussion

### 2.1. PSII-Toxins Conjugates

The two *C. difficile* recombinant toxin fragments, TcdA_B2 and TcdB_GT, derive from a fragment design of TcdA and TcdB assisted by computer modeling. The tripartite organization of both toxins, the GT and CP regions were sub-cloned, and the receptor RBDs were sub-divided into several fragments. On the basis of previous immunological results, we selected the two TcdA_B2 and TcdB_GT fragments as carrier protein for the PSII polysaccharide [19].

PSII is composed of hexaglycosyl repeating units hold together by phosphodiester bonds [22], and the assigned structure has been confirmed by synthesis of the non-reducing end terminal phosphorylated repeating unit [31].

**Figure 1:**
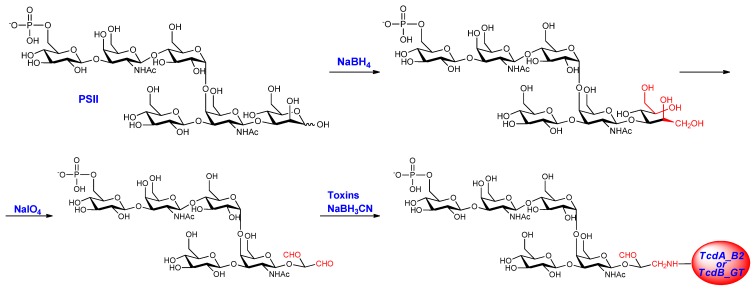
Conjugation scheme of PSII-toxins.

Pure PSII with an average degree of polymerization (avDP) of 15, obtained from fermentation of the R20291 strain (Stoke Mandeville -ribotype 027) as previously reported [23], was conjugated to the two *C. difficile* recombinant fragments derived from TcdA and TcdB after chemical modification of the mannose sugar of the repeating unit at the reducing end. PSII was first reduced with NaBH_4_ and then oxidized with sodium periodate to introduce an aldehyde group useful for the coupling to the lysine residues of the protein by reductive amination [23] (Figure 1).

**Figure 2:**
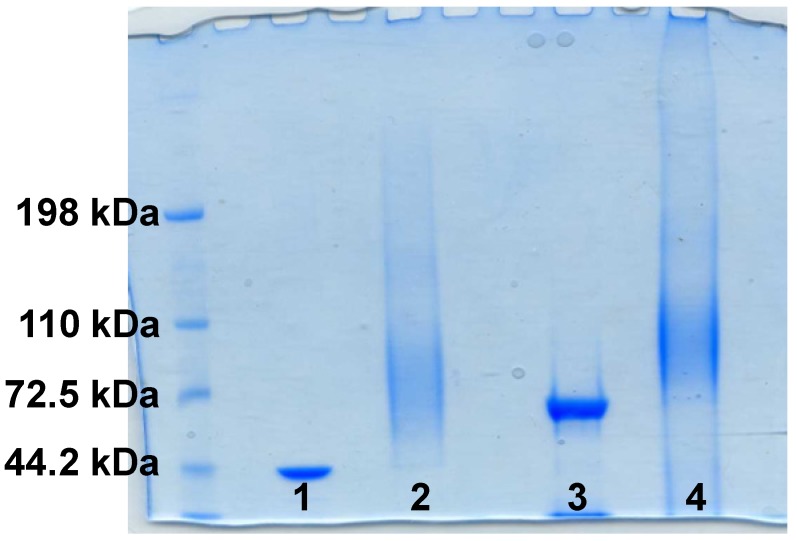
Sodium dodecyl sulfate-polyacrylamide gel electrophoresis (SDS-PAGE) of PSII-toxin conjugates 1: TcdA_B2 fragment protein; 2: PSII-TcdA_B2 conjugate; 3: TcdB_GT fragment protein; and 4: PSII-TcdB_GT conjugate.

The occurrence of complete conjugation was assessed by sodium dodecyl sulfate-polyacrylamide gel electrophoresis (SDS-PAGE), and confirmed by the formation of a broad smear and the concomitant disappearance of the narrow band of the proteins (Figure 2). Subsequently, the glycoconjugates were purified by size exclusion chromatography to remove unbound saccharide and analyzed for their protein content and in terms of total and free saccharide by HPAEC-PAD HPLC as described in literature [23].

Table 1 summarizes the physico-chemical characterization of the purified glycoconjugates in term of total and free saccharide and protein content, compared to the PSII-CRM_197_ conjugate of which the preparation and the characterization were previously reported [23]. Notably, the degrees of glycosylation, ranging from 0.2 to 0.3 (w/w), were comparable for both the products.

**Table 1:** Characteristics of PSII conjugates. avDP: average degree of polymerization.

Conjugates	PSII avDP	Free saccharide (%)	Saccharide/protein (w/w)
PSII-TcdA_B2	15	7.7	0.28
PSII-TcdB_GT	15	22.7	0.33
PSII-CRM_197_ *	21	11.2	0.24

Note: * Characterization previously described [23].

### 2.2. Immunological Evaluation of PSII-Toxins Conjugates

To assess the ability of conjugates to induce anti-PSII antibodies, groups of eight female BALB/c mice were intraperitoneally immunized three times with 2.5 μg carbohydrate based doses of conjugates, at three week-interval between the first and the second dose and two week-interval from the second and the third dose. The conjugates were formulated with the adjuvant MF59, an oil in water emulsion frequently used for seasonal flu vaccination [32]. Adjuvant alone in phosphate buffered saline (PBS) was used as a negative control, while the PSII-CRM_197_ conjugate previously shown capable of inducing a robust anti-polysaccharide immune response [23], and the TcdA_B2 and TcdB_GT fragment proteins already shown to be highly immunogenic [19], were used as a positive control.

Sera obtained after two weeks from the third dose (*post 3 sera*) were analyzed by enzyme-linked immunosorbent assay (ELISA) for their content of anti-PSII and anti-Toxin IgGs. Additionally, the functionality of the anti-Toxin antibodies was investigated *in vitro* to assess the capacity to neutralize the cytotoxicity of TcdA and TcdB.

**Figure 3:**
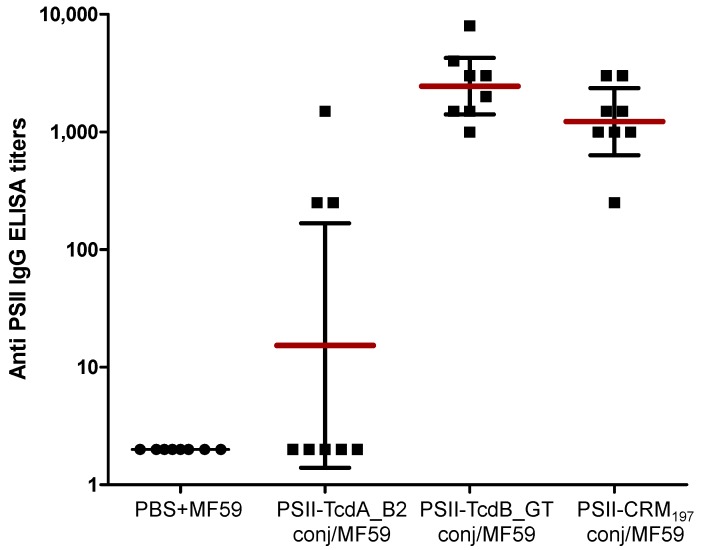
Anti-PSII IgG levels detected in individual *post 3 sera* of BALB/c mice; each dot represents single mouse sera; vertical bars indicate geometric mean titers of each group with 95% statistical confidence intervals as red bars. ELISA: enzyme-linked immunosorbent assay.

The PSII-TcdB_GT conjugate was highly immunogenic, eliciting anti-PSII IgG titres comparable to those obtained with the PSII-CRM_197_ conjugate. Conversely, PSII-TcdA_B2 conjugate induced an anti-polysaccharide response significantly lower than the CRM_197_ conjugate (p 0.006), where IgGs against PSII were induced in three mice only (Figure 3). These results evidenced a better capability of the TcdB_GT fragment protein to function as carrier for the PSII moiety in comparison to the TcdA_B2 peptide.

The antibodies response to the TcdA_B2 and TcdB_GT fragment proteins as carrier was measured and compared with the IgG titers obtained with unconjugated TcdA_B2 and TcdB_GT fragment proteins.

As shown in Table 2, both conjugated toxin fragments evoked a robust anti protein response. However, unconjugated TcdA_B2 elicited significantly higher anti toxin fragment antibodies in comparison to the corresponding PSII conjugate (p 0.002), while TcdB_GT induced IgGs levels comparable to the conjugated form.

**Table 2:** IgG levels detected in individual *post 3 sera* of BALB/c mice against TcdA_B2 and TcdB_GT coating. PBS: phosphate buffered saline.

Antigen	IgG titer (geometric mean titer)	95% confidence interval
PBS	2	-
TcdA_B2 fragment protein	1,024,000	374,868–4,023,000
PSII-TcdA_B2 conjugate	51,499	28,721–109,279
TcdB_GT fragment protein	206,317	47,610–400,390
PSII-TcdB_GT conjugate	106,936	69,489–174,511
PSII-CRM_197_	2	-

**Figure 4:**
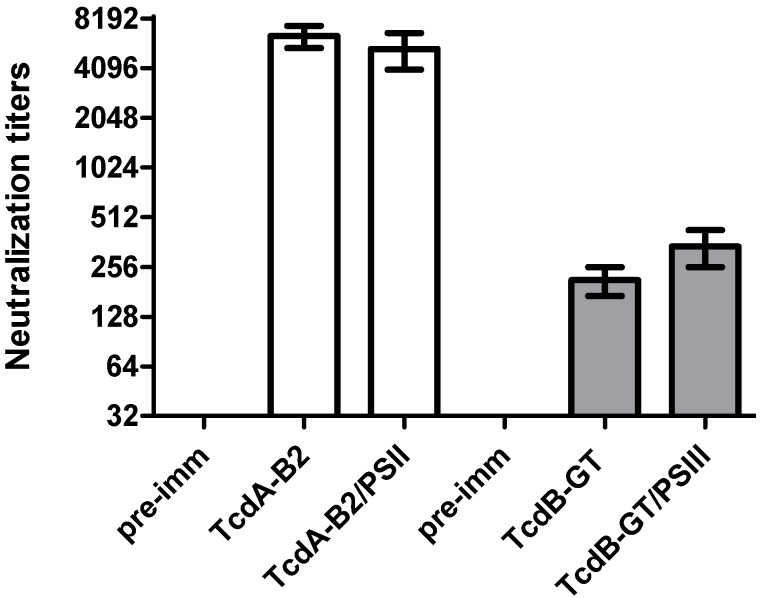
Neutralization titers induced by PSII-conjugated toxin fragments. Sera were collected from mice immunized with TcdA_B2 and TcdB_GT fragments, either alone or conjugated to PSII, in the presence of MF59 adjuvant. Titers were defined as the reciprocal of the highest dilution able to inhibit 100% rounding in IMR-90 human fibroblasts treated with 1 CTU_100_ of toxin A (white column) or B (grey column). Pre-immune sera were used as negative controls. Values reported in the graph represent the mean dilution from three to five independent experiments. The horizontal bars indicate the Standard error.

Next, the functionality of the antibodies was verified by the *in vitro* toxin neutralization assay of pooled sera from mice immunized with the conjugates. Remarkably, as shown in Figure 4, both PSII conjugates elicited toxin neutralizing activity comparable to that induced by their respective protein carrier in unconjugated form, indicating that chemical conjugation did not alter the critical epitopes of recombinant toxin A and B fragments. As previously demonstrated [19], TcdA_B2 and TcdB_GT induce neutralizing antibodies only against the cognate toxin (data not shown), suggesting that vaccination should include combinations of fragments derived from each toxin to achieve the concurrent neutralization of TcdA and TcdB.

## 3. Experimental Section

### 3.1. Preparation and Purification of PSII Glycoconjugates

PSII at avDP 15 was obtained as previously reported in literature. Purified PSII [23] was reduced at the mannose sugar with 50 mM NaBH_4_ (Sigma, St. Louis, MO, USA) in 10 mM sodium phosphate with pH = 9.0 at room temperature for 2 h; the reduced PSII was purified by Sephadex G25 chromatography (G&E Healthcare, Uppsala, Sweden) in water and then oxidized with 15 equivalent of NaIO_4_ (Sigma) in 10 mM sodium phosphate with pH = 7.2 at room temperature for 2 h at the dark. The oxidized PSII was then purified by Sephadex G25 chromatography in water. The oxidized PSII (10 mg/mL) was then conjugated to carrier proteins using a stoichiometry of 4:1 (weight PSII per weight protein) in 200 mM sodium phosphate/1 M NaCl buffer with pH = 8.0, and in presence of NaBH_3_CN (2:1, weight PSII per weight NaBH_3_CN). The mixture was incubated for 48–72 h at 37 °C, mixing very gently with a magnetic stirrer. Conjugates were purified from excess of unconjugated PSII using size exclusion Superdex 75 chromatography (G&E Healthcare) in 10 mM sodium phosphate/10 mM NaCl buffer with pH = 7.2.

### 3.2. Characterization of PSII Glycoconjugates

Conjugates were characterized by SDS-PAGE using 7% Tris-Acetate gels (NuPAGE, Invitrogen, Carlsbad, CA, USA). The samples (5 µg in term of protein) were added of 0.5 M dithiothreitol (1/5 v/v) and NuPAGE LDS sample buffer (1/5 v/v). The mixtures were heated at 100 °C for 1 min. The gel containing loaded samples was electrophoresed at 45 mA in NuPAGE Tris-Acetate SDS running buffer (20×, Invitrogen) and stained with Simply Blue Safe Stain (Invitrogen).

Protein concentration was determined by Micro bicinchoninic acid (BCA) protein assay kit (Thermo Scientific, Rockford, IL, USA). Total saccharide concentration was determined by high performance anionic exchange chromatography-pulsed amperometric detection (HPAEC-PAD) analysis. Unconjugated saccharide was separated by SPE C4 hydrophobic interaction column (0.5 mL resin, Bioselect, Grace Vydac, Columbia, MD, USA) and subsequently estimated by HPAEC-PAD analysis.

### 3.3. Cloning, Expression and Purification of Recombinant Fragments

TcdA_B2 and TcdB_GT were cloned, expressed and purified as previously described [19]. Briefly, fragments were cloned in pet15b+ vector (N-term-His tag) using the Polymerase Incomplete Primer Extension (PIPE) method. The sequences coding for TcdA_B2 (residues 2303–2710 in TcdA) and TcdB_GT (residues 1–543 in TcdB) were amplified by PCR from the *C. difficile* strain 630 genomic DNA and the vector was amplified from the pet15b+ vector; *E.coli* HK100 cells were then transformed with vector/insert hybrids.

PIPE method was employed to generate TcdB_GT (D270A, R273A, Y284A, D286A and D288A) mutant with abrogated enzymatic activity.

The proteins were expressed in *E.coli* BL21 (DE3) cells (Novagen, Darmstadt, Germany) grown in LB. The expression of the protein was induced by addition of 1 mM IPTG to the culture at exponential growth phase and incubation for 4 h at 25 °C.

The protein expression was checked by SDS-PAGE [19].

Recombinant TcdA_B2 and TcdB_GT were purified by immobilized metal ion affinity chromatography (IMAC) and buffer exchange was performed by PD-10 desalting column (G&E Healthcare) or by dialysis. Protein quantification was performed by Micro BCA Protein Assay Kit (Thermo Scientific).

### 3.4. Immunization Protocol

Animal experimental guidelines set forth by the Novartis Animal Care Department were followed in the conduct of all animal studies.

Groups of eight female BALB/c mice (5–6 weeks old) were immunized on days 1, 21 and 35 with 2.5 µg of conjugated carbohydrate antigen formulated with MF59 (mixing equal volume of conjugate and MF59 suspension) as adjuvants. All immunizations were performed by administering a 200 μL of vaccine via intraperitoneal route. Adjuvant alone was used for negative control groups. Sera were collected on days 0 (before the first immunization), 34 and 49 (two weeks after the third immunization).

### 3.5. ELISA Assay

Specific antibodies titers were determined two weeks after the third immunization by ELISA assay. For that purpose 96-well Maxisorp plates (Nunc, Thermo Fisher Scientific, Roskilde, Denmark) were coated with 100 μL/well of 2 μg/mL (protein content) PSII-HSA conjugate (prepared as previously reported [23]) or TcdA_B2 protein or TcdB_GT protein in PBS with pH = 7.2. Plates were incubated over night at 4 °C, then washed three times with TPBS (0.05% Tween 20 in PBS, pH = 7.4) and blocked with 100 μL/well of 3% BSA (Sigma-Aldrich, St. Louis, MO, USA) for 1 h at 37 °C. Each incubation step was always followed by triple TPBS wash. Serum samples were initially diluted 1:1000 in TPBS, transferred into coated-blocked plates (200 μL) and serially two-fold diluted followed by 2 h incubation at 37 °C. Then 100 μL/well of 1:10,000 diluted alkaline phosphatase-conjugated goat anti-mouse IgG (Sigma-Aldrich) were added and left for 1 h at 37 °C. Visualization of bound alkaline phosphatase was performed by adding 100 μL/well of 1 mg/mL para-nitrophenyl-phosphate (pNPP) disodium hexahydrate (Sigma-Aldrich) in 0.5 M diethanolamine buffer with pH = 9.6. After 30 min of development at room temperature, the optical density (OD) of each sample was measured at 405 nm with a microplate spectrophotometer (Biorad). Antibody titres were expressed as the reciprocal of sera dilution corresponding to a cut off OD = 1.0. Each group of immunization was represented as the geometrical mean (GMT) of the single mouse titers.

The statistical and graphical analysis was performed using GraphPad Prism 5 software (GraphPad Software Inc., La Jolla, CA, USA) by applying the Mann Whitney test for statistical analysis.

### 3.6. In Vitro Neutralization Assay

Toxin A and Toxin B were purified from *C. difficile* VPI10463 strain as previously described [19]. IMR-90 human fibroblasts were obtained from American Type Culture Collection (ATCC, Rockville, MD, USA). Cells were grown to 80%–90% confluence in 96-well plates in Eagle’s Minimum Essential Medium (EMEM, ATCC) with 10% fetal calf serum. The minimal doses of Toxin A and Toxin B needed to cause 100% rounding in 24 h (1 CTU_100_) were defined as 20 ng/mL and 10 pg/mL, respectively. For neutralization assay two-fold dilutions of mouse sera from 1:8 to 1:32,000 were pre-incubated with 1 CTU_100_ of each toxin in cell medium for 90 min at 37 °C. Sera and toxins mixtures were then added to the cells and incubated for 16–18 h before analysis. Pre-immune sera were used as negative controls. The endpoint titers were defined as the reciprocal of the highest dilution able to inhibit cell rounding.

## 4. Conclusions

*C. difficile* is considered the most important identifiable cause of healthcare-acquired diarrhea. The tendency of CDI to relapse and lack of efficacious preventative therapies render a vaccine highly recommendable. Typically, the two toxins A and B have been targeted for vaccine development. The surface polysaccharide PSII conjugated to CRM_197_ has also been proven an optimal target for a carbohydrate-based vaccine. In the present study, we explored the feasibility of a glycoconjugate vaccine where the PSII saccharide was conjugated to the two protein fragments TcdA_B2 and TcdB_GT from toxin A and B, respectively.

We demonstrated that TcdB_GT is a very efficient carrier for PSII, since PSII-TcdB_GT was highly immunogenic and induced high titers of anti-polysaccharide IgG antibodies, in a comparable manner to the PSII-CRM_197_ conjugate. On the other hand, the PSII-TcdA_B2 conjugate was less efficient in inducing anti-PSII IgGs. Since the chemical characteristics of the two conjugates are comparable, the reason for this different behavior might reside in the different intrinsic ability of the two carriers to drive the antibody response toward the carbohydrate moiety.

It is important to note that conjugation to the polysaccharide does not impact the neutralizing activity of both TcdA_B2 and TcdB_GT. Therefore, we conclude that conjugation of *C. difficile* carbohydrate antigens to toxin fragments is a promising approach for the design of a conjugate vaccine which targets both surface exposed carbohydrate as well as secreted toxins. Further evaluation in suitable animal models is needed to fully understand the capacity of such constructs to prevent colonization and neutralize toxin activity.
